# Relationship Between Levels of Digital Health Literacy Based on the Taiwan Digital Health Literacy Assessment and Accurate Assessment of Online Health Information: Cross-Sectional Questionnaire Study

**DOI:** 10.2196/19767

**Published:** 2020-12-21

**Authors:** Peggy Liu, Ling-Ling Yeh, Jiun-Yi Wang, Shao-Ti Lee

**Affiliations:** 1 Department of Healthcare Administration Asia University Taichung Taiwan; 2 International Federation for Information Integration Taipei Taiwan; 3 Social Enterprise and Innovation MA Program Graduate School of Humanities and Social Sciences Dharma Drum Institute of Liberal Arts New Taipei City Taiwan; 4 Department of Medical Research China Medical University Hospital China Medical University Taichung Taiwan

**Keywords:** digital health literacy, internet health information, risk group, Taiwan

## Abstract

**Background:**

The increasing amount of health information available on the internet makes it more important than ever to ensure that people can judge the accuracy of this information to prevent them from harm. It may be possible for platforms to set up protective mechanisms depending on the level of digital health literacy and thereby to decrease the possibility of harm by the misuse of health information.

**Objective:**

This study aimed to create an instrument for digital health literacy assessment (DHLA) based on the eHealth Literacy Scale (eHEALS) to categorize participants by level of risk of misinterpreting health information into high-, medium-, and low-risk groups.

**Methods:**

This study developed a DHLA and constructed an online health information bank with correct and incorrect answers. Receiver operating characteristic curve analysis was used to detect the cutoff value of DHLA, using 5 items randomly selected from the online health information bank, to classify users as being at low, medium, or high risk of misjudging health information. This provided information about the relationship between risk group for digital health literacy and accurate judgement of online health information. The study participants were Taiwanese residents aged 20 years and older. Snowball sampling was used, and internet questionnaires were anonymously completed by the participants. The reliability and validity of DHLA were examined. Logistic regression was used to analyze factors associated with risk groups from the DHLA.

**Results:**

This study collected 1588 valid questionnaires. The online health information bank included 310 items of health information, which were classified as easy (147 items), moderate (122 items), or difficult (41 items) based on the difficulty of judging their accuracy. The internal consistency of DHLA was satisfactory (α=.87), and factor analysis of construct validity found three factors, accounting for 76.6% of the variance. The receiver operating characteristic curve analysis found 106 people at high risk, 1368 at medium risk, and 114 at low risk of misinterpreting health information. Of the original grouped cases, 89.6% were correctly classified after discriminate analysis. Logistic regression analysis showed that participants with a high risk of misjudging health information had a lower education level, lower income, and poorer health. They also rarely or never browsed the internet. These differences were statistically significant.

**Conclusions:**

The DHLA score could distinguish those at low, medium, and high risk of misjudging health information on the internet. Health information platforms on the internet could consider incorporating DHLA to set up a mechanism to protect users from misusing health information and avoid harming their health.

## Introduction

The International Telecommunication Union reported in 2019 that over one-half of the world's population was using the internet. In total, 97% of the world’s population lives within reach of a mobile cellular signal and 93% lives within reach of a network that provides a 3G signal or better [1]. In 2014, a European Union report stated that approximately 60% of the public browses the internet every day and 60% of those use the internet to search for health information [2]. More than 70% of US adults used the internet as their primary source of health information in 2019 [3]. In Taiwan, the 2016 Household Digital Opportunity Survey Report [4] concluded that 66.5% of internet users searched for health education, health, or food safety information on the internet [4]. A multinational study by Song et al [5] reported that 51.5% of Americans, 76.9% of South Koreans, and 81.4% of Hongkongers reported using social networking sites to obtain health information, and 66.2%, 94.6%, and 86.1% of them, respectively, reported using content from health information blogs. This implies that people may use the internet to transmit or retrieve health information at any time and anywhere, with the rapid development of digital information tools.

However, Lee et al [6] reported that technology and medical internet rumors accounted for 30% of the total rumor volume. In 2019, a survey in Taiwan found that 26.8% of internet users aged 12 years and older received and forwarded unverified information or news, and 44.9% did not verify information or news [7]. People also tend to believe health information forwarded by friends and relatives because they trust the forwarder [8]. Unnecessary harm could be avoided if people’s digital health literacy could be assessed as a way to understand their capacity to make accurate judgements about online health information. It is hoped that website platforms might then provide warnings about health information that have not been fact-checked or simply do not display that information for people with lower levels of digital health literacy, who are likely to be at high risk of misjudging the accuracy of health information.

The eHealth Literacy Scale (eHEALS) is a subjective measurement that measures perceived skills at finding, evaluating, and employing electronic health information to solve health problems [9,10]. The eHEALS is a widely used measurement tool that has been translated and validated in different languages [11]. Previous studies have used the eHEALS to assess the ability of digital health literacy related to online health information–seeking. Ghaddar et al [12] surveyed online health information–seeking behavior using a questionnaire about patterns of internet use, checking health information on the internet, and knowledge of a particular health information website (Medline Plus [13]). Two studies [14,15] used telephone interviews to ask participants whether they had ever used the internet, their frequency of use, and where they accessed the internet. Diviani et al [16] carried out qualitative in-depth interviews and explored experiences of online health information–seeking and judgment of credibility of online health information. Noblin and Rutherford [17] asked whether participants used a personal health record or other internet-based information system for health care, and whether they were willing to use health care information technology if they did not already do so. These studies, therefore, explored various aspects of online health information–seeking. However, all of the studies used self-reporting, which is regarded as a subjective measurement.

van der Vaart et al [18] explored the association between eHEALS results and an actual performance test lasting up to 1.5 hours. The performance test incorporated operational, formal, information, and strategic internet skills using objective measurements. For example, how to access a health website, save a file, or add a website to the Favorites menu were all operational internet skills [18]. A study by Quinn et al [19] investigated the relationship between health literacy, eHealth literacy, and actual online health information–seeking behavior by integrating software into a web browser to objectively monitor online interaction; the data were coded for analysis. The study participants were recruited from a convenience sample of 54 university students and staff, and they completed the search tasks under laboratory conditions with 6 health questions about diabetes, obesity, influenza, nutrition, and analgesic medication. These studies, therefore, used objective measures, but their designs were not directly comparable with real-world situations because of participants’ characteristics or the limited health questions or time required. To the best of our knowledge, no study has yet reported on the relationship between the risk of misinterpreting health information, measured as level of digital health literacy, and ability to make accurate judgments about online health information, especially using a wide range of health information and a large number of participants with real-world scenarios.

Therefore, this study aimed to develop an instrument for digital health literacy assessment (DHLA) based on the eHEALS. It distinguished the different risk levels for digital health literacy using DHLA scores by assessing the accuracy of judgments about internet health information resembling that found in the real world.

## Methods

We developed an instrument called the DHLA, which drew from the eHEALS. An online health information bank with right and wrong answers was also constructed to check the ability of the DHLA to discriminate between people at low, medium, and high risk of misinterpreting health information using receiver operating characteristic curve (ROC) analysis. This study was reviewed and approved by the regional review board in central Taiwan (approval number CRREC-108-096).

### Study Participants

This study’s participants were Taiwanese residents aged 20 years and older. Snowball sampling was used to identify potential participants among colleagues, friends, and family members of the researchers in northern, central, and southern Taiwan. A total of 350 participants were recruited, to maximize the range of socioeconomic status. They were asked to send the online survey to their friends and family members. Email and online communications software were used to send quick response codes or website links to participants to enable them to complete the online questionnaire.

### Instruments

The eHEALS includes 8 questions that use a 5-point Likert-type scale, with scores ranging from 1 (strongly disagree) to 5 (strongly agree). This generates a single factor. The total score ranges from 8 to 40 points. A higher score shows better digital health literacy [9,10]. A previous study [20] showed that the eHEALS structure varies with the environment and setting, and researchers have also recommended that the scale should consider environmental and cultural issues [21,22]. In Taiwan, Wu [23] used Yahoo data to establish that almost 15% of items of health information obtained during searches related to folklore and custom. An example item was that “smokers should eat pig’s blood to cleanse their lungs.” Therefore, 2 questions were added in this study. Question 1 asked participants to rate their ability to use information technology (“I am able to use a computer/smartphone to find information that I need on the internet”), and question 10 asked about their belief in local folk medicine (“I feel confident about the health care information based on folklore and customs that I found on the internet”). The responses to the questions directly rated the level of ability and belief in the question content, instead of rating attitudes with levels of agreement. Questions 1 to 6 involved self-assessment of digital health literacy with responses from 1 (very poor) to 5 (very good). Questions 7 to 9 were concerned with how convincing people found internet health information from different sources, and question 10 was about trust in health information from folklore and customs. The responses ranged from 1 (not at all convincing) to 5 (very convincing). The 10 items of the DHLA are shown in Multimedia Appendix 1.

### Construction of the Online Health Information Bank

This study constructed a scenario in which internet users could complete an assessment of their digital health literacy using the DHLA before starting their search for health information. This would allow the website to identify their level of digital health literacy. Users with a lower level of digital health literacy would then see a warning message alongside unverified items of health information, or simply not see that kind of health information. Thus, this study involved a search of items of health information from the internet in the real world. The health sectors in Taiwan set up a platform to conduct fact-checking of health information issued by people on the internet. Items of health information were obtained from the Fact Check Column on the Taiwan Food and Drug Administration website [24] and the Healthcare Fact Check Column on the Health Promotion Administration website [25]. These sites carry information about food safety, drugs, medical devices, cosmetics, medical treatment, preventative health, disease screening, health promotion, and general medicine. Any resident of Taiwan can check this health information via an internet search. The health authority invites experts to meet and develop a consensus about whether the information is right or wrong, with a rationale. The content of items of health information and expert feedback were obtained from public government information platforms [26,27]. The online health information bank in this study focused on food safety, drugs, medical treatment, preventative health, disease screening, health promotion, and general medicine, which is very similar to the range of health information available on the internet.

We carried out a two-stage content validity check to construct the online health information bank. A total of 600 items of health information were included from the two public sector platforms, which was reduced to 529 after repeated items were removed. In stage 1, four medical experts were invited to classify how difficult it was to judge the accuracy of each piece of content and feedback from the public platforms. The items were classified into categories as easy, moderate, or difficult to judge, defined as items for which more than 80%, 50%, and less than 30% of the public, respectively, were able to determine credibility. In stage 2, one public health expert and three members of the public were invited to discuss the expert difficulty categorizations. The group meeting considered that the items of health information must be stated precisely, and that highly specialized items of health information were not appropriate. Therefore, items of health information were excluded if experts had judged them to be only partially correct, or where determining credibility required knowledge of chemical composition and its effects. This resulted in an online health information bank containing 310 items, of which 147 were easy, 122 were moderate, and 41 were difficult. Sample items and difficulty levels are included in Multimedia Appendix 2.

### Data Collection

The questionnaire was completed anonymously via an online questionnaire platform. The questionnaire introduced the DHLA, then asked for basic information such as sex, age group, education level, marital status (married or not), employment, income, whether their job was health-related, quantity (level) of internet use, self-reported health, height, weight, waist circumference, exercise frequency, and place of residence. Finally, the system randomly selected 5 items (2 easy, 2 moderate, and 1 difficult) from the online health information bank for participants to consider. For each item, participants were asked to determine whether the information was right or wrong, with an option for unsure. The questionnaire platform set standard answers, and participants were given a score of 1 when the answer given was correct (accurate judgment), and 0 when they answered incorrectly or were not sure (not accurate judgment). Items were selected at random because real-world health information is varied and has diverse levels of difficulty. This approach therefore simulated a real-world search for health information on the internet. Once the participant had completed the questionnaire, the platform showed them the correct answers to the 5 items for their future reference, as an important ethical issue.

### Analysis

The reliability and internal consistency of the DHLA was assessed using Cronbach alpha and item-scale correlation. The validity was assessed using convergent validity and construct validity. Spearman correlations were used between total DHLA scores and age, education level, and level of internet use. In line with previous studies, we hypothesized negative correlations with age and positive correlations with education level and level of internet use, and these were used to investigate the convergent validity [16,18]. Principal component analysis with promax rotation was used for construct validity, assuming that there were mild correlations among the factors, and eigenvalues greater than 1 were used as an indication of how many factors to retain. Distributional properties of the DHLA were investigated by examining the normality of the total scores and detecting floor and ceiling effects. Skewness and kurtosis values below −1 or above 1 were considered to show non-normality [28]. Floor or ceiling effects were considered present if more than 15% of participants scored either the worst or the best possible score on the DHLA [29].

We used ROC analysis to distinguish high-, medium-, and low-risk groups using different cutoff points for the DHLA score, based on the participants’ ability to make an accurate judgment of easy, moderate, and difficult items. A cutoff value for DHLA scores was determined from the point on the ROC curve. In general, the cutoff point is based on an area of more than 50% under the curve. We used the Youden index to select the most appropriate cutoff value: (sensibility + specificity – 1) is maximum [30].

Descriptive statistics were used to demonstrate the proportion of correct answers, with means and standard deviations of the DHLA scores in different risk groups. Chi-square tests were used to detect the relationship between difficulty levels of the health information items and the risk groups. Analysis of variance (ANOVA) was used to investigate the differences in the DHLA score for each factor across different risk groups. Posthoc analysis was used to test whether the differences among risk groups were statistically significant following ANOVA. We used discriminate analysis on the three risk groups to explore the probability of correctly classifying people by their DHLA scores. Convergent validity was tested using Spearman correlation analysis to examine the relationship between difficulty levels of items of health information and risk groups, in terms of age, education level, and level of internet use.

In the last stage of data analysis, the chi-square test was used to analyze the relationship between digital health literacy and individual attributes, self-reported health status, and level of internet use, followed by logistic regression analysis to analyze factors related to risk group identified by DHLA scores. The high-risk group was compared with the medium-risk group and with a combined medium- and low-risk group. SPSS statistical software (version 22.0; IBM Corp) was used for statistical analysis. *P* values <.05 were considered statistically significant.

## Results

### Participants

A total of 1871 online questionnaires were collected, of which 1588 were valid after excluding those that were not complete. In total, 63.7% (1011/1588) of respondents were women, 40.6% (645/1588) were aged 45 to 63 years, 87.7% (1393/1588) had an education level of college (959/1588, 60.4%) or above (434/1588, 27.3%), and 57.8% (918/1588) were married. A total of 46.7% (742/1588) of the participants were professionals, and 36.9% (586/1588) worked in the service industry; 36.1% (573/1588) had a monthly income of New Taiwan dollar (NT) $30,000 to $50,000 (US $1049 to US $1748.34), 91.1% (1447/1588) browsed the internet several times every day, and 46.3% (736/1588) perceived that their health was good. Finally, 15.6% (247/1588) exercised every day and 20.9% (332/1588) did not exercise at all.

### Distributional Properties

The total DHLA scores were approximately normally distributed, with a skewness of −0.53 and a mean score of 35.50 (SD 5.56). Nobody obtained the worst possible score (ie, 10) and 5 (5/1588, 0.3%) participants scored the maximum possible score (ie, 50), so the floor and ceiling effects were acceptable.

### Reliability and Validity

We used principal component factor analysis on DHLA scores to give three factors. These three factors were digital health literacy (questions 1 to 6), belief in medicine (questions 7 to 9), and belief in folk remedies (question 10), and they accounted for 76.6% of the variance, with eigenvalues of 4.87, 1.68, and 1.11, respectively. Table 1 shows the factor loadings for the 10 items, ranging from 0.63 to 0.93. The standardized Cronbach alpha was .87 for the whole scale, .92 for the subscale on digital health literacy, and .77 for the subscale on belief in medicine. Thus, the internal consistency overall was good. All item-total correlations were also statistically significant, ranging from 0.27 to 0.87. If an item was deleted, Cronbach alpha ranged from .84 to .89.

**Table 1:** Digital health literacy assessment (DHLA) internal reliability and factor structure.

				Factor loading									
				Domain name^a^									
DHLA item		Mean (SD)		1		2		3		Item-scale correlation, *P*<.001		Cronbach alpha if item deleted	
Full scale		35.50 (5.56)										.87	
1	My ability to use computer/smartphone to find information that I need on the internet		4.04 (0.91)		0.77						0.73		.85
2	My ability to find health- or disease-related information on the internet		3.89 (0.86)		0.91						0.85		.84
3	My ability to find information on internet to understand health problems or diseases		3.82 (0.86)		0.93						0.87		.84
4	My ability to find information on the internet to answer the questions on health care or disease treatment		3.67 (0.88)		0.91						0.85		.84
5	My ability to use information found on the internet to discuss with health care professionals		3.27 (0.99)		0.79						0.75		.85
6	My ability to judge whether the health care information found on the internet is accurate or not		3.45 (0.88)		0.82						0.78		.85
7	Beliefs about the health care information that I find on the internet		3.31 (0.64)				0.63				0.53		.87
8	Beliefs about the health care information provided by physicians that I find on the internet		3.60 (0.66)				0.90				0.56		.86
9	Beliefs about the health care information provided by hospitals that I find on the internet		3.82 (0.67)				0.89				0.48		.87
10	Beliefs about the health care information based on folklore and customs that I find on the internet		3.37 (0.79)						0.90		0.27		.89

^a^Domain names are as follows: 1=digital health literacy, 2=belief in medicine, 3=belief in folk remedies.

Table 2 shows the convergent validity using Spearman correlations between total DHLA scores and the variables of age, education level, and level of internet use. All correlation analyses were statistically significant, and the correlation coefficients showed mild correlation (<0.3) with age (ρ=–0.19, *P*<.001), education (ρ=0.22, *P*<.001), and level of internet use (ρ=0.17, *P*<.001).

**Table 2:** Spearman correlation analysis of total scores for the DHLA and scores for accurate judgment across different difficulty levels and risk groups.

Characteristic	DHLA^a^ (ρ)	DHLA (*P* value)		Difficulty level scores								Total score (ρ)	Total score (*P* value)	Risk group (ρ)	Risk group (*P* value)
				Easy^b^			Moderate^c^		Difficult^d^						
				ρ	*P* value		ρ	*P* value	ρ		*P* value				
Age (≤34 to ≤65 years)	−0.19	<.001		0.02	.322		0.03	.268	0.02		.344	0.04	.168	−0.18	<.001
Education level (junior high school or below to university or above)	0.22	<.001		0.05	.196		0.05	.031	0.10		<.001	0.09	<.001	0.18	<.001
Level of internet use (never to several times per day)	0.18	<.001		0.04	.245		0.07	.007	0.07		.006	0.08	<.001	0.21	<.001

^a^DHLA: digital health literacy assessment.

^b^Pearson correlation between DHLA and easy level=0.04.

^c^Pearson correlation between DHLA and moderate level=0.07, *P*=.009.

^d^Pearson correlation between DHLA and difficult level=0.17, *P<*.001.

### Defining Risk Group

ROC analysis was used to analyze the scores for each factor of the DHLA to identify the cutoff points between the tendency to answer correctly and incorrectly. As shown in Table 3, there were good cutoff points at 15.5, 8.5, and 1.5 for digital health literacy, belief in medicine, and belief in folk remedies, respectively. The area under the curve was less than 0.50 for all difficulty levels for the factor belief in folk remedies, so a score of less than the cutoff value (1.5) was defined as the tendency to answer correctly. However, for the other two factors (digital health literacy and belief in medicine), less than the cutoff value (15.5 and 8.5, respectively) was defined as the tendency to answer incorrectly. The new scores for the three DHLA factors were therefore defined as 1 (tends to be incorrect) or 2 (tends to be correct) based on the ROC cutoff points. Participants were considered to be at low risk of misjudgments if they scored 2 for all three factors, at medium risk if they scored 2 for two of the three factors, and at high risk if one or none of the three factor scores was 2. This gave a total number of 106 participants in the high-risk group, 1368 participants in the medium-risk group, and 144 participants in the low-risk group.

**Table 3:** Receiver operating characteristic curve analysis of the cutoff points of the digital health literacy assessment scores by accurate judgment and difficulty level.

Domain name and difficulty level			Cutoff		Sensitivity		Specificity		AUC^a^		95% CI
**Digital health literacy**											
	Easy	15.5		0.95		0.09		0.49		0.45-0.54	
	Moderate	15.5		0.95		0.09		0.54		0.50-0.57	
	Difficult	15.5		0.96		0.08		0.56		0.53-0.59	
**Belief in medicine**											
	Easy	8.5		0.96		0.03		0.52		0.47-0.56	
	Moderate	8.5		0.97		0.05		0.50		0.47-0.54	
	Difficult	8.5		0.97		0.04		0.53		0.50-0.56	
**Belief in folk remedies**											
	Easy	1.5		0.91		0.07		0.48		0.44-0.52	
	Moderate	1.5		0.91		0.10		0.46		0.43-0.50	
	Difficult	1.5		0.91		0.08		0.48		0.46-0.53	

^a^AUC: area under the curve.

Table 4 shows that 86.9% of easy items, 78.3% of moderate items, and 54.2% of difficult items were judged correctly. The highest proportion of correct answers was always in the low-risk group (90.4%, 79.8%, 57.0%), then the medium-risk group (87.0%, 78.9%, 55.0%), and finally the high-risk group (82.1%, 68.9%, 41.5%). There was no statistically significant relationship between correctly assessing the easy items and risk group (*P*=.190). However, there was a statistically significant relationship with risk group for both the moderate and high difficulty items (*P*=.049; *P*=.023). The mean DHLA score in the low-risk group (mean 39.32, SD 4.72) was greater than in the medium- (mean 36.84, SD 4.37) and high-risk groups (mean 25.31, SD 5.04), and this difference was statistically significant. The three factors of digital health literacy, belief in medicine, and belief in folk remedies showed very similar results. For the pair comparison of the post hoc test, the mean scores for digital health literacy and belief in medicine for the low-risk group were both greater than for the medium-risk group, but this difference was not statistically significant. The discriminant analysis showed that 89.6% of the original grouped participants were correctly classified.

**Table 4:** Comparison of proportion of accurate judgments of online health information sources and digital health literacy assessment (DHLA) scores across different risk groups.

Difficulty level/DHLA		Risk group										
		High (n=106)	Medium (n=1368)	Low (n=114)		Total (n=1588)		χ²/F (*df*)		*P* value		Posthoc test
**Difficulty level (correct answer, %)**												
	Easy	82.1	87.0	90.4		86.9		3.37 (2)		.190		
	Moderate	68.9	78.9	79.8		78.3		6.05 (2)		.049		
	Difficult	41.5	55.0	57.0		54.2		7.57 (2)		.023		
**DHLA score**												
	Mean	25.31	36.84	39.32				360.12 (2)		<.001		H^a^<M^b^, H<L^c^, M<L; *P*<.001
	SD	5.04	4.37	4.72								
**Digital health literacy score**								303.77 (2)		<.001		
	Mean	13.14	22.75	23.29								H<M, H<L; *P*<.001
	SD	4.60	3.82	4.13								
**Belief in medicine**								92.36 (2)		<.001		
	Mean	8.78	10.86	11.04								H<M, H<L; *P*<.001
	SD	2.38	1.46	1.44								
**Belief in folk remedies**								388.63 (2)		<.001		
	Mean	3.39	3.23	5.0								H<M, *P*=.02; H<L, M<L, *P*<.001
	SD	0.82	0.66	0.0								
**Predicate group^d^**												
	High	56	50	0								89.6% of original grouped cases correctly classified
	Medium	1	1367	0								
	Low	0	114	0								

^a^High-risk group.

^b^Medium-risk group.

^c^Low-risk group.

^d^Standardized canonical discriminant function: Wilks lambda=0.688, *P*<.001; eigenvalue=0.454; canonical correlation=0.559.

This study also examined the convergent validity of scores of difficulty level and risk groups in Table 2. The Spearman correlation coefficients across difficulty levels showed a very mild correlation with education level (ρ=0.05–0.10) and level of internet use (ρ=0.04–0.07). The correlations with moderate and difficult items were statistically significant. The Pearson correlation coefficient between the DHLA score and the score in each difficulty level ranged from 0.4 to 0.1 and was statistically significant for moderate and difficult items. The scores for easy items across risk groups were not statistically significant. The correlation coefficients between risk groups and age, education level, and level of internet use indicated a mild but statistically significant correlation (ρ=−0.18 to 0.21). There was a negative correlation with age, and positive correlations with levels of education and internet use.

### Description and Associated Factors Analysis in Different Risk Groups

Table 5 shows that gender, age, education level, marital status, employment, income, level of internet use, self-reported health status, and exercise habits were statistically significantly associated with risk group. Overall, 70.7% (75/106) of the subjects aged 45 years and older were in the high-risk group, and 28.9% (33/114) were in the low-risk group. In total, 55.7% (59/106), 89.5% (1224/1368), and 96.5% (110/114) of participants in the high-, medium-, and low-risk groups, respectively, had a college degree or higher. This shows that higher levels of education were associated with lower risk of misinterpreting health information. Participants who worked as professionals and in the service industry were mostly in the medium- and low-risk groups (703/742 [94.7%] and 556/586 [94.9%]). Participants who worked as laborers accounted for 16.0% (17/106), 6.9% (95/1368), and 2.6% (3/114) of the high-, medium-, and low-risk groups, respectively. Participants who worked in the service industry accounted for 28.3% (30/106), 37.6% (514/1368), and 36.8% (42/114) of participants in the high-, medium-, and low-risk groups, respectively. The proportion of participants with an income of NT $50,000 (US $1748.34) or greater per month was 28.3% (30/106) in the high-risk group, 37.6% (514/1368) in the medium-risk group, and 39.4% (45/114) in the low-risk group, indicating that a higher income is associated with a lower proportion of participants in the high-risk group. The proportion of participants who never or only rarely browsed the internet accounted for 28.3%, 2.5%, and 0% of the high-, medium-, and low-risk groups. A high proportion of the low-risk group (70/114, 61.4%) had good health, and the proportion with poor health in the high-, medium-, and low-risk groups was 17.0% (18/106), 6.6% (90/1368), and 3.5% (4/114). This suggests that most participants with poor health were in the high-risk group. The proportion of participants who did not exercise was higher in the higher risk groups (27/106 [25.5%], 286/1368 [20.9%], and 19/114 [16.7%] in the high-, medium-, and low-risk groups, respectively).

**Table 5:** Risk groups and characteristic distribution of study participants.

Characteristic		Risk group, n (%)					
		High (n=106)	Medium (n=1368)	Low (n=114)	Total, n (%)	χ² (*df*)	*P* value
**Sex**						17.5 (2)	<.001
	Male	46 (43.4)	471 (34.4)	60 (52.6)	577 (36.3)		
	Female	60 (56.6)	897 (65.6)	54 (47.4)	1011 (63.7)		
**Age (years)**						142.6 (6)	<.001
	≤34	20 (18.9)	364 (26.6)	52 (45.6)	436 (27.5)		
	35–44	11 (10.4)	398 (29.1)	29 (25.4)	438 (27.6)		
	45–64	49 (46.2)	564 (41.2)	32 (28.1)	645 (40.6)		
	≥65	26 (24.5)	42 (3.1)	1 (0.9)	69 (4.3)		
**Education level**						234.5 (6)	<.001
	Junior high school or below	21 (19.8)	9 (0.7)	0 (0)	30 (1.9)		
	Senior high school	26 (24.5)	135 (9.9)	4 (3.5)	165 (10.4)		
	University or college	45 (42.5)	841 (61.5)	73 (64)	959 (60.4)		
	Graduate school	14 (13.2)	383 (28)	37 (32.5)	434 (27.3)		
**Married**						7 (2)	.029
	No	40 (37.7)	569 (41.6)	61 (53.5)	670 (42.2)		
	Yes	66 (62.3)	799 (58.4)	53 (46.5)	918 (57.8)		
**Employment**						40.8 (6)	<.001
	Professional	39 (36.8)	653 (47.7)	50 (43.9)	742 (46.7)		
	Service industry	30 (28.3)	514 (37.6)	42 (36.8)	586 (36.9)		
	Manual work	17 (16.0)	95 (6.9)	3 (2.6)	115 (7.2)		
	Unemployed	20 (18.9)	106 (7.7)	19 (16.7)	145 (9.1)		
**Income (NT $)^a,b^**						90.3 (12)	<.001
	0	22 (20.8)	86 (6.3)	15 (13.2)	123 (7.7)		
	≤$23,800	20 (18.9)	79 (5.8)	18 (15.8)	117 (7.4)		
	$23,801-$30,000	10 (9.4)	167 (12.2)	9 (7.9)	186 (11.7)		
	$30,001-$50,000	24 (22.6)	522 (38.2)	27 (23.7)	573 (36.1)		
	$50,001-$70,000	12 (11.3)	276 (20.2)	18 (15.8)	306 (19.3)		
	$70,001-$100,000	9 (8.5)	148 (10.8)	20 (17.5)	177 (11.1)		
	≥$100,001	9 (8.5)	90 (6.6)	7 (6.1)	106 (6.7)		
**Level of internet use**						177.8 (4)	<.001
	Never or rarely	30 (28.3)	34 (2.5)	0 (0.0)	64 (4)		
	Several times per week/month	5 (4.7)	70 (5.1)	2 (1.8)	77 (4.8)		
	Several times per day	71 (67.0)	1264 (92.4)	112 (98.2)	1447 (91.1)		
**Health status**						29.4 (4)	<.001
	Poor	18 (17.0)	90 (6.6)	4 (3.5)	112 (7.1)		
	Normal	51 (48.1)	649 (47.4)	40 (35.1)	740 (46.6)		
	Good	37 (34.9)	629 (46)	70 (61.4)	736 (46.3)		
**Exercise frequency**						11.8 (4)	.019
	Never	27 (25.5)	286 (20.9)	19 (16.7)	332 (20.9)		
	Sometimes	54 (50.9)	883 (64.5)	72 (63.2)	1009 (63.5)		
	Daily	25 (23.6)	199 (14.5)	23 (20.2)	247 (15.6)		

^a^A currency exchange rate of NT $1=US $0.035 is applicable.

^b^Average regular earnings of all employees (including full-time or part-time employees with Taiwanese nationality or foreigners) was New Taiwan dollar (NT) $42,495. The poverty level income ranged from NT$11,648 to NT$24,293 in 2019 in Taiwan.

Table 6 shows that participants at high risk of misjudging health information had a lower education level, lower income, and poor health, and they did not browse or only rarely browsed the internet. These differences were statistically significant. The probability of participants in the medium-risk group having an education level of senior high school, college, or graduate school was 4.65, 13.22, and 18.40 times, respectively, compared with participants in the high-risk group. Participants with an income were more likely to be part of the medium-risk group than those without any income, with an odds ratio (OR) of 1.76 (95% CI 0.58-5.36) to 4.23 (95% CI 1.56-11.47). Participants with good health were more likely to be in the medium-risk group (OR 1.93, 95% CI 0.90-4.14) than those with poor health (OR 2.98, 95% CI 1.30-6.83). Compared with those who were never or only rarely online, those who were online several times every day or several times every week/month were more likely to be in the medium-risk group (OR 3.80, 95% CI 1.71-8.46 and OR 9.19, 95% CI 2.61-32.37, respectively). Similar results were obtained when the high-risk group was compared with the two lower risk groups.

**Table 6:** Logistic regression analysis across different risk groups (*P*<.001).

Characteristic		Medium-risk versus high-risk^a^, OR^b^ (95% CI)	Medium- and low-risk versus high-risk^c^, OR (95% CI)
**Education level (ref: junior high school or less)^d^**			
	Senior high school	4.65 (1.54-13.99)	4.50 (1.50-13.52)
	University/college	13.22 (4.24-41.23)	13.46 (4.32-41.93)
	Graduate school	18.40 (5.03-67.36)	19.11 (5.23-69.90)
**Income (New Taiwan $) (ref: $0)^e^**			
	≤$23,800	0.90 (0.37-2.20)	0.93 (0.38-2.28)
	$23,801-$30,000	4.06 (1.44-11.45)	3.86 (1.37-10.87)
	$30,001-$50,000	4.08 (1.72-9.69)	3.78 (1.59-8.95)
	$50,001-$70,000	4.23 (1.56-11.47)	3.92 (1.45-10.59)
	$70,001-$100,000	2.18 (0.75-6.33)	2.16 (0.75-6.23)
	≥$100,001	1.76 (0.58-5.36)	1.62 (0.54-4.90)
**Level of internet use (ref: never/rarely)**			
	Several times per week/month	9.19 (2.61-32.37)	9.05 (2.57-31.84)
	Several times per day	3.80 (1.71-8.46)	3.95 (1.77-8.79)
**Health status (ref: poor)**			
	Normal	1.93 (0.90-4.14)	2.00 (0.93-4.31)
	Good	2.98 (1.30-6.83)	3.22 (1.40-7.39)

^a^–2 log-likelihood: 564.251; Nagelkerke *R^2^*: 0.311.

^b^OR: odds ratio.

^c^–2 log-likelihood: 576.339; Nagelkerke *R^2^*: 0.309.

^d^ref: reference.

^e^A currency exchange rate of NT $1=US $0.035 is applicable.

## Discussion

### Principal Results

This study developed the DHLA instrument, drawing from the eHEALS, and carried out a reliability and validity analysis. The study also constructed an online health information bank from public sector sources to simulate information likely to be found by real-world users searching for online health information. The study used ROC analysis to find DHLA cutoff values to classify participants into high-, medium-, and low-risk groups based on their likelihood of misinterpreting health information. A higher DHLA score was associated with a lower risk of health information misjudgment. High-risk participants tended to have a low education level, low income, and poor health, and never or rarely browsed the internet for information. The proportions of accurate judgments on moderate and difficult items of health information were lower in the high-risk group (68.9% and 41.5%, respectively) by approximately 10% to 15% compared with the medium-risk (78.9% and 55.0%, respectively) and low-risk (79.8% and 57.0%, respectively) groups. The difference between risk groups was less among the items that were considered easy. The instrument therefore seems to distinguish more accurately between the high-risk and low/medium-risk groups than between the low- and medium-risk groups.

### Limitations

The first limitation of this study is that the sample does not represent the population in Taiwan. We did our best to recruit participants across the widest possible range of socioeconomic status, including from among those using social welfare, via colleagues, friends, and relatives. The participants covered different age groups, genders, income levels, and education levels. However, the participants tended to have a high education level, which might have affected their digital health literacy. The second limitation is the data collection mechanism (an online questionnaire platform), which cannot reach participants who are unfamiliar with the internet or mobile devices. However, a previous study found that there were no significant differences in eHEALS scores between paper-based and online questionnaires [31]. In the future, telephone or paper-based channels could be considered for data collection, to broaden the ranges of scores for digital health literacy. This might increase the differences in DHLA scores between the medium- and low-risk groups. The third limitation was the online health information bank, which included 310 items of health information across different difficulty levels (147 easy, 122 moderate, and 41 difficult). This was more items than were used in previous studies [12,18,19]. We considered that the majority of people needed to be able to judge the accuracy of health information from the online health information bank, so we excluded any items regarded as requiring professional knowledge. This may have limited the number of difficult items to 41 and might have made it harder to differentiate between medium- and low-risk groups.

### Comparison with Prior Work

The eHEALS is unidimensional, with a Cronbach alpha of 0.88, whereas the DHLA has three dimensions, with a Cronbach alpha of .87. Using a criterion of 0.70 to 0.90 as a measure of good internal consistency [29], the DHLA has adequate internal consistency. Item-scale correlation of the DHLA had a range of 0.27 to 0.87, which was satisfactory because the coefficients were greater than 0.20 [32] for all items. Except for one item on folk remedies (0.27), all the others were over 0.48. The DHLA was developed from the eHEALS, adding two more items and changing the format of response. This gave a different factor structure. Abma et al [33] reported that their relative correlation size of convergent validity ranged from 0.2 to 0.4 with mild to moderate correlation, although the expected direction of the correlation—positive or negative—is also important. DHLA scores showed a negative correlation with age and a positive correlation with education level and level of internet use. The weak correlation coefficient was statistically significant around 0.20, so the convergent validity was considered acceptable.

In a systematic review, Diviani et al [34] found that 10 (26%) of the 38 studies examined the relationship between health literacy and capacity to assess online health information. However, only five studies used the eHEALS as a measure to explore the issues related to online health information searching. The Newest Vital Sign (Pfizer Inc) screening tool has been used in several studies as a performance test to assess the ability to make accurate judgments about online health information [12,17,35]. This tool is based on a nutrition label composed of six questions and requires 3 minutes to complete [36]. A study by Quinn et al [19] developed six health questions as a performance test, including strategic areas such as diabetes, obesity, influenza, nutrition, and analgesic medication. Several studies have used performance tests to collect data, but these take considerable time to complete, often around 1.5 hours [18,37,38]. Studies using performance tests therefore often limit the areas of health information covered, and so do not reflect real-world situations. Taking a long time to collect data might lower the validity of studies because of participants’ fatigue. In this study, the majority of participants took approximately 6 minutes to assess five items of health information randomly selected from the online health information bank. The items in the health information bank were drawn from real-world items on the internet. The online health information bank is thus considered to be a reasonable analogy to online health information, but it cannot be regarded as a performance test comparable to those used in previous studies [12,17,18,35,37,38].

The online health information bank contained approximately 300 items, giving it both quantity and variety, which might have resulted in the weak positive correlation coefficients found between difficulty levels and levels of education and internet use (Table 2). Nevertheless, the correlation coefficients were statistically significant, except for easy items. The difficulty levels were categorized as items for which more than 80% (easy), 50% (moderate), and less than 30% (difficult) of people are able to determine credibility. In the study, however, the proportions of correct answers were 86.9% (easy), 78.3% (moderate), and 57.0% (difficult), which was obviously higher than the definitions. This might be because 96.5% of participants had at least a university or college education. However, this study used the health information bank and the DHLA score to distinguish between those at high, medium, and low risk of misinterpreting information by levels of digital health literacy. The study results revealed that the high-risk group had the lowest proportion of correct answers when judging the health information items. This means that the DHLA could be used by health information platforms on the internet as a way to identify—and therefore protect—users at high risk of misinterpreting health information.

Systematic reviews have found that education level is related to digital health literacy [39]. Neter and Brainin [15] studied adults aged 18 years and older and found that digital health literacy was not related to gender or self-reported health, but was associated with age, education level, diseases and conditions, and level of internet use. Xesfingi and Vozikis [40] found that digital health literacy was related to age, education level, and exercise frequency. Del Giudice et al [41] showed that digital health literacy was associated with age, education level, self-reported health, and level of internet health information use. We compared the high-risk group with (1) the medium-risk group, and (2) the combined medium- and low-risk groups, and found that distinguishing factors included education, income, level of internet use, and self-reported health status. Therefore, the factors found in this study are very similar to factors found in previous studies on digital health literacy [15,39-44].

Del Giudice et al [41] found that education level had a weak positive correlation (ρ=0.11, *P*=.001) with digital health literacy. Their sample was divided into two groups: those studying or working in the health sector, and those who were not. Interestingly, in our study, having a health-related job was not statistically significantly correlated with different risk groups. We found that higher education level (ρ=0.22, *P*<.001) was associated with lower risk of health information misjudgment, showing better ability to judge credible health information. This finding suggests that people with a low education level may need help to improve their digital health literacy to avoid misuse of online health information and its potentially negative effects on their health.

Choi and Dinitto [42] demonstrated that people with a low level of education had poor digital health literacy compared with the US population as a whole. A systematic literature review reported an association between income and internet access and digital health literacy [39]. We found that participants with an income of NT $23,800 to $70,000 (US $832.20 to $2447.65) were better able to judge the credibility of internet health information than those with no income. However, the ORs of participants with an income of NT $70,001 (US $2447.68) and NT $100,000 (US $3496.64) or greater was lower than the OR of those with an income of NT $23,801 (US $832.24) to $70,000 (US $2447.65) (2.18 and 1.76, respectively, versus approximately 4; Table 6). This suggests that the relationship between income level and risk group based on DHLA scores may be an inverted U shape. It is possible that those with an income of NT $70,000 (US $832.20) or greater were more cautious in selecting health information and did not want to give incorrect answers, and were thus more likely to respond “unsure,” which resulted in a lower probability of being in the medium- and low-risk groups.

Studies have shown that self-reported health status is significantly correlated with digital health literacy [41,45,46]. We found that participants with poor health were more likely to be in the high-risk group, with low digital health literacy. It is therefore important to help those with poor health status to improve their health literacy through health education. This will reduce their risk of using false information from the internet.

Zrubka et al [44] indicated that many studies found an association between digital health literacy and level of internet use, although some studies have found that the correlation is only weak [18]. We found that participants who go online several times per day, per week, or per month were better able to identify correct health information and were at a lower risk of health information misjudgment than those who rarely or never use the internet. However, those who used the internet several times per week or month had the highest probability of being in the medium- or low-risk groups (OR 9.19, 95% CI 2.61-32.37), followed by those using the internet several times per day (OR 3.80, 95% CI 1.71-8.46), compared with those who never or rarely used the internet. It seems likely that those who browse the internet several times per day might become blasé about the quality of the information available, whereas those who use the internet several times per week or month may be more careful. It may be helpful to improve internet access for people who never or rarely use the internet and encourage them to use it more often. However, websites may also need to provide a mechanism to protect them from misinterpreting information.

### Conclusions

Our results showed that DHLA scores could distinguish between those at low, medium, and high risk of misjudging health information on the internet. Health information platforms on the internet could therefore use the DHLA to set up mechanisms to protect users from misusing health information and thereby avoid harming their health. This would mean incorporating the DHLA into the website and verifying it with a larger sample. Simultaneously, this approach may decrease the possibility that users will receive erroneous health information that could threaten their quality of life.

## Appendix

Multimedia Appendix 1 Digital health literacy assessment.

Multimedia Appendix 2 Sample items of online health information bank.

## Abbreviations

ANOVA: analysis of variance
DHLA: digital health literacy assessment
eHEALS: eHealth Literacy Scale
NT: New Taiwan dollar
OR: odds ratio
ROC: receiver operating characteristic
