# Supplementary material for: Relationship Between Levels of Digital Health Literacy Based on the Taiwan Digital Health Literacy Assessment and Accurate Assessment of Online Health Information: Cross-Sectional Questionnaire Study
Source: J Med Internet Res. 2020 Dec 21;22(12):e19767. doi: 10.2196/19767 (PMC7781799; doi:10.2196/19767)
Supplement: Multimedia Appendix 1 [file jmir_v22i12e19767_app1.docx]

**Appendix:** Digital Health Literacy Assessment

| In English | In Tradition Chinese |
| --- | --- |
| 1. I am able to use a computer/smartphone to find information that I need on the internet. | 1. 我操作電腦/智慧型手機上網搜尋我所需資料的能力。 |
| 2. I am able to find health or diseases related information on the internet. | 2. 我上網搜尋健康或疾病相關資訊的能力 |
| 3. I am able to find information on internet to understand health problems or diseases. | 3. 我上網找資料瞭解健康問題或疾病的能力 |
| 4. I am able to find information on the Internet to answer the questions on healthcare or disease treatment. | 4. 我上網找資料解答健康照護或疾病治療問題的能力 |
| 5. I am able to use information found on internet to discuss with healthcare professionals. | 5. 我利用上網找到的資訊跟醫護人員討論的能力 |
| 6. I am able to judge whether the health care information found on internet is accurate or not. | 6. 我判斷上網找到健康照護資訊正確與否的能力 |
| 7. I feel confident about the Healthcare information that I find on the internet. | 7. 我對於上網找到的健康照護相關資料 |
| 8. I feel confident about the healthcare information provided by physicians that I found on the Internet. | 8. 我對於上網找到醫師提供的健康照護相關資訊 |
| 9. I feel confident about the healthcare information provided by hospitals that I found on the Internet. | 9. 我對於上網找到醫院提供的健康照護相關資訊 |
| 10. I feel confident about the healthcare information based on folklore and customs that I found on the Internet | 10 我對於上網找到與民間習俗有關的健康照護相關資訊 |
| Q1 to Q6 were rated as Very poor(1), poor(2), Neutral(3), Good(4), Very Good(5) ;  Q7 to Q10 were rated as not at all convinced(1), Not convinced(2), Neutral(3), convinced(4), very convinced (5) | 第1-6題評分為非常不好(1),不好(2),普通(3),好(4),非常好(5)  第7-10題評分為非常不相信(1),不相信(2),普通(3),相信(4),非常相信(5) |
