# Supplementary material for: Relationship Between Levels of Digital Health Literacy Based on the Taiwan Digital Health Literacy Assessment and Accurate Assessment of Online Health Information: Cross-Sectional Questionnaire Study
Source: J Med Internet Res. 2020 Dec 21;22(12):e19767. doi: 10.2196/19767 (PMC7781799; doi:10.2196/19767)
Supplement: Multimedia Appendix 2 [file jmir_v22i12e19767_app2.docx]

Appendix : Sample items of online health information bank.

1. Smokers can eat more pig blood, which will cleanse their lungs. (rating easy)

2. I have been working all day long, so I don't have to go out for exercise. (rating easy)

3. It is helpful to drink bone broth, which can supplement calcium. (rating moderate)

4. Applying sesame oil the whole body, which can repel mosquitoes. (rating moderate)

5. Smoking e-cigarettes is not addictive. (rating difficult)

6. For emergency treatment of patients with myocardial infarction, immediately tap the inner side of the elbow. (rating difficult)
